# Biotremology in arthropods

**DOI:** 10.3758/s13420-020-00428-3

**Published:** 2020-07-06

**Authors:** Sofia Cividini, Giuseppe Montesanto

**Affiliations:** 1 Department of Biostatistics, Institute of Translational Medicine, University of Liverpool, Crown Street, Liverpool, L693BX UK; 2 Dipartimento di Biologia, Università degli Studi di Pisa, Pisa, Italy

**Keywords:** Animal communication, Behavioral processes, Substrate-borne signals, Vibrational communication, Insects, *Armadillo officinalis*

## Abstract

Effective communication is essential in animal life to allow fundamental behavioral processes and survival. Communicating by surface-borne vibrations is likely the most ancient mode of getting and exchanging information in both invertebrates and vertebrates. In this review, we concentrate on the use of vibrational communication in arthropods as a form of intraspecific and interspecific signaling, with a focus on the newest discoveries from our research group in terrestrial isopods (Crustacea: Isopoda: Oniscidea), a taxon never investigated before in this context. After getting little attention in the past, biotremology is now an emerging field of study in animal communication, and it is receiving increased interest from the scientific community dealing with these behavioral processes. In what follows, we illustrate the general principles and mechanisms on which biotremology is based, using definitions, examples, and insights from the literature in arthropods. Vibrational communication in arthropods has mainly been studied in insects and arachnids. For these taxa, much evidence of its use as a source of information from the surrounding environment exists, as well as its involvement in many behavioral roles, such as courtship and mating, conspecific recognition, competition, foraging, parental care, and danger perception. Recently, and for the first time, communication through surface-borne waves has been studied in terrestrial isopods, using a common Mediterranean species of the Armadillidae family as a pilot species, *Armadillo officinalis* Duméril, 1816. Mainly, for this species, we describe typical behavioral processes, such as turn alternation, aggregation, and stridulation, where vibrational communication appears to be involved.

**Electronic supplementary material:**

The online version of this article (10.3758/s13420-020-00428-3) contains supplementary material, which is available to authorized users.

Animal communication is a dynamic system where there is always an individual that transmits a signal (the sender) and an individual that may interpret this signal correctly and modify its behavior consequently (the receiver) (Alcock, 2009; Greenfield, 2010; Hill, 2009; Hill & Wessel, 2016; Krams, 2010; Markl, 1983; McGregor, 2005; McGregor & Peake, 2000). The emitted signal can be visual, chemical, tactile, acoustic, or vibrational and received through different sensory systems (sight, smell, taste, touch, hearing) or specific receptors (Hill, 2009; Hill & Wessel, 2016). An efficient communication allows fundamental behavioral processes in animal life (e.g., conspecific recognition, courtship and mating, parental care, competition, foraging, coordination of group behavior), as well as defense and survival strategies (Borgia, 1985; McGregor, 2005; McGregor & Peake, 2000; Yorzinski, 2017). All this happens inside a very complex network of environmental signals and information exchanges coming from many different transmitters and receivers (Alcock, 2009; Greenfield, 2010; Krams, 2010; McGregor, 2005; McGregor & Peake, 2000). Single individuals can publicly exchange information to many receivers, obtain and use information from other individuals’ private communications, or alter their private communication when other receivers are nearby (Greenfield, 2010). The concept of communication network arose from the observation that signals emitted by animals travel well over the space between transmitter and receiver. That makes them easy to intercept by other individuals, the so-called eavesdroppers (Alcock, 2009; Greenfield, 2010; Hill & Wessel, 2016; Krams, 2010; McGregor & Peake, 2000; Peake, 2005). Interceptive eavesdropping is a widely spread form of interspecific communication. The most studied and known of which mainly interests heterospecific alarm signals, even among quite different and not related species (Alcock, 2009; Greenfield, 2010; Hill & Wessel, 2016; Krams, 2010; Peake, 2005; Virant-Doberlet, Kuhelj, Polajnar, & Šturm, 2019). Moreover, inside this complex network of signaling, every individual can play both the role of the sender/receiver and the role of the eavesdropper (Greenfield, 2010; Hill & Wessel, 2016). All these aspects of animal communication are present and play an essential role in both vertebrate and invertebrate behavioral patterns and dynamics (Ball, 2009; Bishop, Denton, Pomeroy, & Twiss, 2015; Hill & Wessel, 2016; Klump, 2009; Krams, 2010; McGregor, 2005; Virant-Doberlet et al., 2019).

In what follows, we turn attention to a new, emerging field of study in animal communication—biotremology—which is receiving increased interest from the scientific community dealing with these behavioral processes. Notably, in this review, we concentrate on the studies of biotremology aimed to elucidate behavioral processes and communication mediated by surface-borne vibrations in arthropods, also highlighting the recent discoveries concerning vibrational communication in terrestrial isopods. This taxon was never studied before in this context. We thus focus on *Armadillo officinalis*—studied by our research group—as a pill bug species particularly interesting for its ability to produce stridulations and the high sensitivity to substrate-borne vibrations.

## Biotremology: A vibratory exchange of information

The world of arthropods is as fascinating as complex and mysterious. These tiny animals have impressive characteristics and capabilities, and understanding how they communicate and interact among them represents a compelling challenge. For instance, it is astonishing how numerous species of insects can perceive, distinguish, and manage substrate-borne vibrations, produced by their conspecifics, or other animals, for multiple aims. Caterpillars *Semiothisa aemulataria* Walker, 1861 can identify their predators—wasps or stink bugs from birds or herbivores—thanks to perception and qualitative and quantitative differentiation of surface-borne vibrations that these insects produce while foraging on a leaf (Castellanos & Barbosa, 2006). Similarly, dry wood termites of the species *Cryptotermes secundus* Hill, 1925 can distinguish the vibrational signals produced by their conspecifics from those made by their more numerous and stronger competitors living in the same tree—the subterranean species *Coptotermes acinaciformis* Froggatt, 1898. In this way, they can avoid a direct, likely lethal, clash (Evans et al., 2009).

Communication through surface-borne vibrations does not necessarily imply the use of sensory systems (such as sight and hearing) to function and is widely used by arthropods. This form of vibrational communication is the object of the study of biotremology, although this discipline also includes other behaviors guided by substrate vibrations (Hill, Virant-Doberlet, & Wessel, 2019).

Biotremology has many unique characteristics, but also characteristics shared with other disciplines (Hill et al., 2019). So, scientists are currently still discussing terms and definitions. We point out that some behavioral dynamics described in the following sections, such as predator–prey interactions, are not part of the classical communication signal theory paradigm. Indeed, predators and prey do not use strategies that define a classical communication system (Hill et al., 2019). Nevertheless, biotremologists include the study of predators and prey in biotremology because of the intrinsic use of vibrational behavior (Hill et al., 2019). “*This new knowledge from outside the communication paradigm can be used within the paradigm after it has been discovered*, page 21” (Hill et al., 2019). For this reason, we do not separate the two aspects as part of the same discipline.

### What is biotremology?

According to the definition recently proposed by Hill and Wessel (2016), biotremology is “*the study of mechanical communication by surface-borne* waves page R189”. (Hill et al., 2019). This form of communication is one of the most ancient and widespread in both invertebrates and vertebrates (Cocroft, Gogala, Hill, & Wessel, 2014; Hildebrand, 1995; Hill, 2001, 2008, 2012; Hill et al., 2019; O’Connell-Rodwell, Hart, & Arnason, 2001). The use of substrate-borne vibrations in animal communication doubtless goes back much earlier than the use of air-borne waves. Nevertheless, most of the scientists have started studying and dedicating their attention to this phenomenon, particularly in the past three decades (Hildebrand, 1995; Hill, 2001, 2008, 2009, 2012; Virant-Doberlet & Čokl, 2004). Indeed, as reported by Hill (2012), the scientific community had long argued that the inelasticity of substrates and the high magnitude of propagation speeds and wavelengths involved could not allow substrate-borne vibrations to transfer biologically useful information among animals (Schwartzkopff, 1974). Mainly, if animals were tiny, substrate-borne waves could only alert them of a disturbance in the offing (Schwartzkopff, 1974). The surfaces through which vibrations propagate can be highly variable, such as the ground, the surface of the water, a leaf, a spider web, or a honeycomb (Hill, 2009). These vibrations can be perceived with specific sensory systems or receptors (see Table 1; Keil, 1997, 2001) that are able to measure the oscillations at the boundary between media (Hill et al., 2019; Hill & Wessel, 2016).

**Table 1.** Mechanoreceptors in insects and other invertebrates (Keil, 1997, 2001)

Mechanoreceptor	Response	Description	Species	Location
*Bristle type*	Touch	These mechanoreceptors consist of a hair jointed to the cuticle through an elastic fibrillar membrane and transmit deflection of their distal part to a sensory dendrite. They respond when a direct touch occurs.	Flies	Thorax, head, neck region, wings, interfacetal hairs on the eyes
			*Rhodnius* ^[1]^	Antennae
			Locusts ^[2]^	Head
			Honeybees ^[3,4]^	Neck region
*Trichobothrium or filiform type*	Faint air currents Low-frequency sounds Vibrations	Like Bristles, filiform mechanoreceptors consist of a hair jointed to the cuticle through an elastic fibrillar membrane and transmit deflection of their distal part to a sensory dendrite. Well-characterized and studied in crickets, they are involved in the localization of stimuli regarding, for example, a predator approaching from behind, trigging the escape response. In spiders, they are known as *trichobothria* and used in prey detection.	Crickets Cockroaches ^[5,6]^ Other Orthopterans Lepismatids ^[7]^	Cerci Cerci Cerci Cerci
			Caterpillars ^[8]^	Trunk
			Bugs ^[1,9,10]^	Trunk, antennae
			Spiders ^[11-13]^ Scorpions ^[14, 15]^ Pseudo-scorpions Mites	Legs and pedipalps Pedipalps Pedipalps Body and tarsi
			Soil-dwelling arthropods as Symphylans, Pauropods, and Diplurans ^[16,17]^	
*Campaniform type*	Cuticle deformation	These mechanoreceptors consist of a cuticular dome and respond to stress and deformations in the body wall.	Crickets	Cerci, closed to leg joints
			Dipterans ^[18]^ Strepsipterans ^[19]^	Wing bases
*Scolopidial type*	Stretch	The mechanoreceptors of this type assist in the detection of mechanical stress, are inside the body, and often are involved in hearing.	Mosquitoes ^[20]^	Johnston’s organ
			Crickets ^[21-23]^ Locusts Other insects	Ears Ears Ears

*Note.* See Supplementary for references.

[1] McIver & Siemicki, 1984; [2] Smola, 1970; [3] Lindauer & Nedel, 1959; [4] Thurm, 1965; [5] Camhi, 1980; [6] Gnatzy, 1976; [7] Berg, 1994; [8] Tautz, 1977, 1978; [9] Draslar, 1973; [10] Gaffal, 1976; [11] Christian, 1971; [12] Görner, 1965; [13] Görner & Andrews, 1969; [14] Hoffmann, 1967; [15] Messlinger, 1987; [16] Haupt, 1970, 1978; [17] Bareth & Juberthie-Jupeau, 1986; [18] Voelker, 1982; [19] Pix et al., 1993; [20] Risler, 1977; [21] Autrum, 1942; [22] Autrum & Schneider, 1948; [23] Gray, 1960

Communication mediated by surface-borne vibrations is an essential channel of information exchange among many animal species, both when used alone and in combination with other modes of communication, such as visual, tactile, olfactory, hearing signals, or pheromones (Cocroft et al., 2014; Hill, 2001, 2008, 2009, 2012, 2015; Hill & Wessel, 2016). Moreover, surface-borne vibrations are involved both in intraspecific and in interspecific communication as a possible option of a multimodal signaling strategy (Barth, 1997; Claridge, 1985; Čokl & Virant-Doberlet, 2003; Hill, 2008, 2009; Hill & Wessel, 2016). Vibrational communication assists both invertebrates and vertebrates to retrieve information from the surrounding environment and is used in multiple contexts (Barth, Bleckmann, Bohnenberger, & Seyfarth, 1988; Cocroft et al., 2014; Cocroft & Rodríguez, 2005; Hill, 2008; Meyhofer, Casas, & Dorn, 1997; Pfannenstiel, Hunt, & Yeargan, 1995; Sandeman, Tautz, & Lindauer, 1996; Virant-Doberlet & Čokl, 2004). It has been estimated that a few hundred thousand invertebrate species (insects, arachnids, crustaceans, worms) use surface-borne vibrations as a primary form of communication (Cocroft & Rodríguez, 2005; Hill, 2008, 2009, 2012; Virant-Doberlet & Čokl, 2004). Indeed, communication by surface-borne waves may also assist in courtship and mating, competition, localization of conspecifics, parental care, foraging, and danger perception (Caldwell, Johnston, McDaniel, & Warkentin, 2010; Castellanos & Barbosa, 2006; Cocroft, 1996, 1998, 1999, 2001; Elias, Mason, & Hoy, 2004; Evans et al., 2009; Gogala, Čokl, Drašlar, & Blaževic, 1974; Hebets, Elias, Mason, Miller, & Stratton, 2008; Hill, 2001, 2008, 2009, 2019; Hill et al., 2019; Hill & Wessel, 2016).

### Communication via surface-borne waves and the definition of “active space”

Communication via surface-borne vibrations consists of a complex network of signaling involving conspecifics, heterospecifics, rivals, and exploiters (Cocroft et al., 2014; Cocroft & Rodríguez, 2005; McVean & Field, 1996; Stewart & Zeigler, 1984; Virant-Doberlet et al., 2014). This form of communication can extend to up many meters, even for the smallest arthropods. Still, mechanism efficacy depends on several factors, such as the amplitude of transmitted signals from the sender, their attenuation, filtration, or alteration during propagation through the substrate and the sensitivity of the receiver (Čokl & Virant-Doberlet, 2003; Cocroft et al., 2014; Cocroft & Rodríguez, 2005; Cocroft, Shugart, Konrad, & Tibbs, 2006; Endler, 1993; Eriksson, Anfora, Lucchi, Virant-Doberlet, & Mazzoni, 2011; Hill et al., 2019; McVean & Field, 1996; Michelsen, Fink, Gogala, & Traue, 1982; Miklas, Stritih, Čokl, Virant-Doberlet, & Renou, 2001; Mortimer, 2017; Stewart & Zeigler, 1984; Virant-Doberlet et al., 2014).

In acoustic communication, sounds move in a homogeneous enough medium, such as air or water. Conversely, the quality of vibratory communication and information perceived by animals like arthropods depends on the nature of the substrate through which vibrational signaling goes through and on the background noise (Cocroft et al., 2014; Cocroft & Rodriguez, 2005; Čokl & Virant-Doberlet, 2003; Hill, 2008; Hill et al., 2019; Mazzoni, Eriksson, Anfora, Lucchi, & Virant-Doberlet, 2014; Mortimer, 2017). A discontinuity present in the substrate, such as the gap between leaves, seems, however, not to be a limitation on the communication range of vibrational signals (Eriksson et al., 2011).

Based on the definition introduced by Mazzoni et al. (2014), an “*active space*, page 127” represents the space where animals can efficiently exchange information through vibrational signals. The active space is generally variable in extension, and it may be limited by physical constraints, such as filtering of frequency, damping or energy loss, and distortion of the temporal pattern of the propagating vibration (Cocroft et al., 2014; Hill et al., 2019; Mortimer, 2017). Vibrational energy decreases during propagation through the substrate because of friction, and damping, distortion, and filtering are mainly related to the type of waves and the properties and geometry of the substrate (Cocroft et al., 2014; Cocroft & Rodríguez, 2005; Čokl & Virant-Doberlet, 2003; Hill et al., 2019; Kolsky, 1964; Mortimer, 2017). For this reason, substrate-borne vibrations are not generally pure tones (i.e., tones with a sinusoidal waveform and unique frequency), as containing complex oscillatory patterns with many frequencies simultaneously (narrowband or broadband vibrations) (Mortimer, 2017). These vibrational signals are nonstationary signals because their frequency content changes with time. So, the pattern of vibrational signals inside an active space can be irregular with a nonmonotonic decreasing of amplitude (namely, not following a single direction, but increasing and decreasing on different intervals of wavefunction’s domain) (Čokl, 1988; Čokl, Zorovic, & Millar, 2007; Mazzoni et al., 2014).

For many arthropods, the active space is generally restricted to the host plant (where an animal lodges and subsists) or parts of the host plant (Mazzoni et al., 2014). The extension of the active space network useful for signaling can also suffer reductions because of additional environmental factors. In this event, the receiver can no longer detect the signal emitted by the sender because it is masked by background noise or made unreliable by nontarget individuals or species (Mazzoni et al., 2014). For instance, insects like treehoppers (*Tylopelta gibbera*) and leafhoppers (*Scaphoideus titanus*) use specific signals to disrupt or jam courtship of a rival male (Legendre, Marting, & Cocroft, 2012; Mazzoni et al., 2014; Mazzoni, Prešern, Lucchi, & Virant-Doberlet, 2009).

In biotremology research, both frequency and temporal patterns of magnitude are essential factors to consider (Mortimer, 2017). Indeed, these provide complementary information to animals, allowing them to distinguish between biotic (living or once-living organisms) or abiotic (nonliving physical and chemical elements) sources and discriminate among different species (Barth et al., 1988; Mortimer, 2017; Schmitt, Schuster, & Barth, 1994). Some insects such as honeybees, bumblebees, stingless bees, and some groups of flies can produce low-frequency substrate-borne vibrations using their thoracic flight muscles, or, for honeybees, by tremulation of the abdomen (Hill, 2008, 2015; Kirchner, 1997; Lewis & Schneider, 2000; Sandeman et al., 1996). A recent study by Davranoglou, Cicirello, Taylor, and Mortimer (2019) demonstrated, for instance, that the planthopper *Agalmatium bilobum* (Fulgoromorpha: Issidae) uses fast, cyclical abdominal motions to generate substrate-borne vibrations. This mechanism allows it, despite its small size, to transmit efficiently pulsing signals containing a broad spectrum of frequencies through the substrate, which makes its vibrational communication effective.

### Biotremology in terrestrial isopods

Vibrational communication with the related behavioral patterns is prevalently known and studied in insects and arachnids. Conversely, it is much less known and understudied in other species of arthropods, for instance, in terrestrial isopods, in which it plays a not less important role. Mainly, some species of terrestrial isopods belonging to the roller-type—that is, able to roll up on themselves—are fascinating from a vibrational communication perspective because they are equipped with a stridulatory apparatus. The latter allows them to produce stridulations in determined circumstances, as we illustrate in this review.

Among these stridulating, roller-type terrestrial isopods, *Armadillo officinalis* mostly stands out for its sensitivity to substrate-borne vibrations. The species owes its name to its alleged pharmaceutical properties. Indeed, in the past, after being dried and pulverized, it was used to facilitate diuresis and digestion (Duméril, 1816). Both biology and ethology of *A. officinalis* are still little known. Nevertheless, the study of this species could offer broad-spectrum insights on the communication mechanisms and behavioral processes mediated by substrate-borne vibrations, which may be generalizable also to other, more studied classes of arthropods.

Currently, to our knowledge, we are the first investigators to study the aspects of biotremology in *A. officinalis*, as a pilot species of the Armadillidae family (Isopoda: Oniscidea). We thus introduce this terrestrial isopod with its main distinctive features. In the following paragraphs, we describe the newest vibrational communication discoveries concerning it, found by our research group.

#### *Armadillo officinalis*: A pill bug producing stridulations

Terrestrial isopods, commonly known as pill bugs, slaters, or woodlice, are generally part either of mesofauna or macrofauna and play a species-specific role in the decomposition of leaf litter (Abd El-Wakeil, 2015; Zimmer, Pennings, Buck, & Carefoot, 2002). Most terrestrial isopods feed on detritus or plants, and animals, both alive and dead (Warburg, 1993), but they are also coprophagic animals (Drobne, 1995; Hassall & Rushton, 1982; Ullrich, Storch, & Schairer, 1991).

Belonging to the family Armadillidae, *A. officinalis* Duméril, 1816 is a common species of terrestrial isopod that has adapted to live in xeric environments populated by various types of vegetation (Messina, Montesanto, Pezzino, Caruso, & Lombardo, 2011; Messina et al., 2014; Messina, Pezzino, Montesanto, Caruso, & Lombardo, 2012), in the Mediterranean basin and on the western coasts of the Black Sea (Schmalfuss, 1996, 2003). Typically, *A. officinalis* can live on different substrates, namely sand, silty-clay substrates, or rocks. These pill bugs have mainly nocturnal habits (Vandel, 1962). In the daytime, they remain under stones or other shelters, forming quite large aggregates. It is thought that aggregation is likely useful for preventing both desiccation and predation, as reported for many terrestrial isopods (Broly, Deneubourg, & Devigne, 2013; Broly, Devigne, Deneubourg, & Devigne, 2014; Broly, Mullier, Deneubourg, & Devigne, 2012). *A. officinalis* is an iteroparous species—that is, producing offspring more times in a lifetime—and the reproductive period depends on the particular region to which it belongs—for instance, from June to August in France (Vandel, 1962), mainly in October in Israel (Warburg, 2013), and from May to July in Sicily (Messina et al., 2011; Messina et al., 2012).

This terrestrial isopod species can produce stridulations using a ledge of scales situated on the propodus of the fourth and fifth pereopods (Caruso & Costa, 1976; Taiti, Paoli, & Ferrara, 1998). The ability to produce stridulations was first described by Verhoeff (1908) after breeding in captivity some specimens of *A. officinalis* collected in Sicily. Subsequently, only a preliminary study on stridulation in *A. officinalis* was published in a local Italian journal (Caruso & Costa, 1976). A similar stridulatory organ was also observed in *Cubaris everesti* Vandel, 1973 from Nepal (Taiti et al., 1998), as well as in two other undetermined species belonging to the same genus that belongs to the same family of *Armadillo* (S. Taiti, personal communication).

**Fig. 1:**
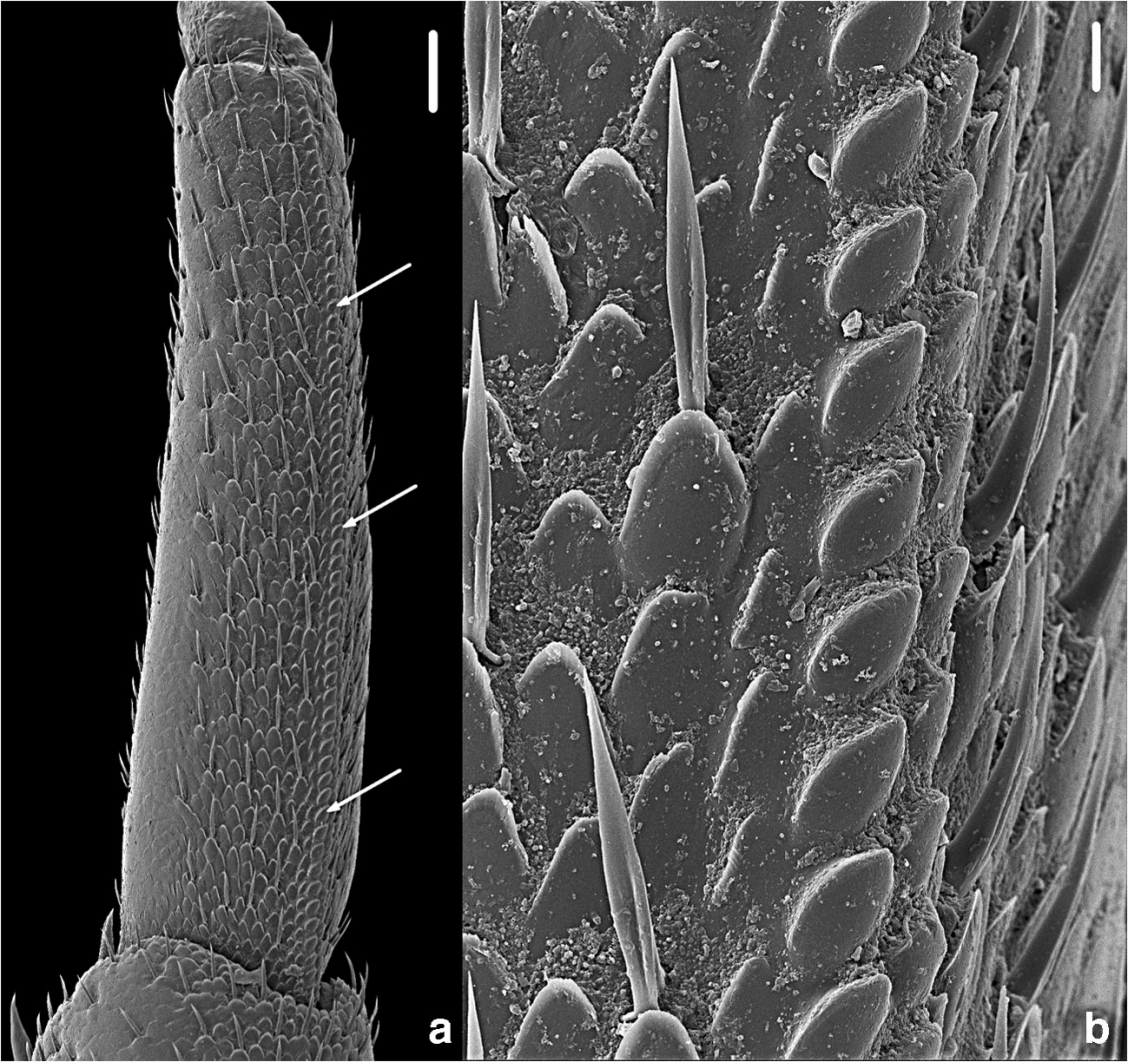
***A****rmadillo officinalis* Duméril, 1816. An adult female from Fiesole, Tuscany, Italy. Scanning electron microscope magnification of the stridulatory apparatus. **a** Sternal view of the propodus of pereopod 4, showing the line of scales of semicircular plates (scale bar: 100 μm). **b** Detail of the line of scales (scale bar: 10 μm)

The stridulatory apparatus of *A. officinalis* consists of a crest, situated on the propodus of the fourth and fifth pair of legs (see Fig. 1a). It is formed of more than 60 semicircular plates placed as a rack and perpendicular to the central axis of the propodus (see Fig. 1b) (Caruso & Costa, 1976). This crest overlaps the median line of the tergal part, increasing its protrusion and forming the so-called “*plectrum*, page 19” (sensu Caruso & Costa, 1976). It has been supposed that the surface on which the “*plectrum*, page 19” can rub (the so-called “*pars stridens*, page 19”) should match the free part of the inner face of the epimera of the fifth, sixth, and perhaps of the seventh pereonite (Caruso & Costa, 1976).

**Fig. 2:**
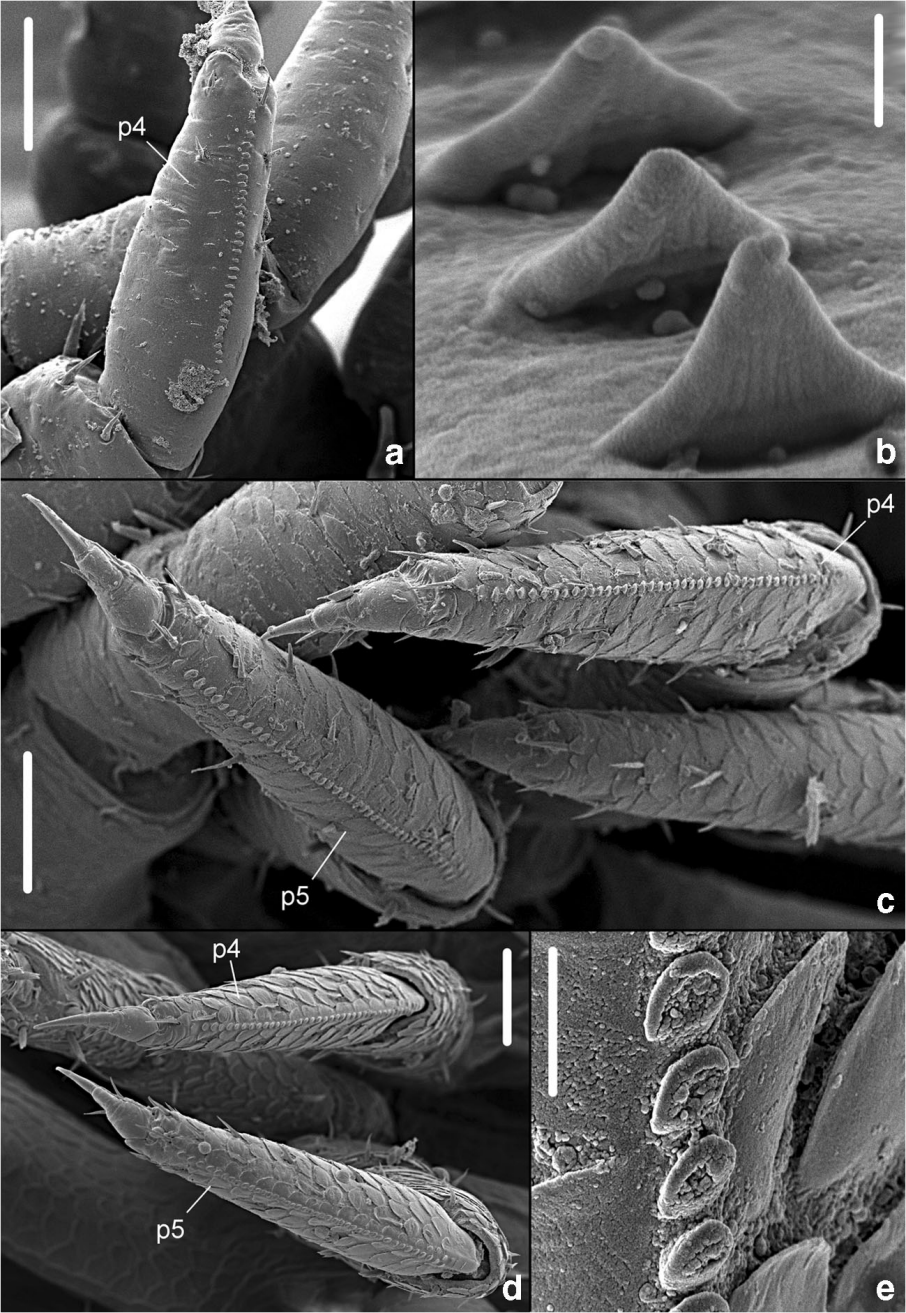
*Armadillo officinalis* Duméril, 1816. Manca stages M I–M III from Catania, Sicily, Italy. Scanning electron microscope magnification of the stridulatory apparatus. a Sternal view of the propodus of pereopod 4 (p4), in M I (scale bar: 50 μm). b Scales on the propodus of pereopod 5, in M I (scale bar: 1 μm). c The line of scales on the propodus of pereopod 4 (p4) and pereopod 5 (p5), in M II (scale bar: 50 μm). d The line of scales on the propodus of pereopod 4 (p4) and pereopod 5 (p5), in M III (scale bar: 50 μm). e Scales on the propodus of pereopod 4, in M III (scale bar: 5 μm)

Recently, Montesanto (2018) studied post marsupial manca stages—equivalent to larval stages in insects—in *A. officinalis* intending to detect in which period of development the stridulatory apparatus (SA) of this terrestrial isopod begins forming. According to Montesanto’s observations, *A. officinalis* exhibits three stages concerning post marsupial manca (M): M I, M II, and M III. The stridulatory apparatus is present from these early stages of development. In stage M I, the SA consists of a line formed by 28–30 scales (plectrum) on the sternal margin of the propodus of the fourth and fifth pereopods (see Fig. 2a–b). In stage M II, the SA increases in dimension, reaching a length equal to 38–40 scales, having a circular shape (see Fig. 2c). In stage M III, no further dimensional increase in the SA was observed (see Fig. 2d–e) (Montesanto, 2018).

## Behavioral processes and vibrational communication in arthropods

In the literature, many studies have demonstrated the involvement of the mechanisms of communication through substrate-borne vibrations in numerous behavioral processes and adaptive behaviors in arthropods. In the last years, we have seen an increase in the number of published articles from many research groups worldwide on this intriguing topic, by also involving new taxa, never considered before. That confirms a consistently higher interest of the scientific community for vibrational communication in animal behaviors, and specifically in arthropods, where it seems to play a crucial role in many behavioral patterns and dynamics.

In what follows, we focus on the aspects of vibrational communication at the level of vital behaviors in arthropods, such as courtship and mating, recognition of conspecifics, predation, defense strategies, eavesdropping, foraging, and parental care. Furthermore, we analyze in detail the discoveries from our research group concerning substrate-borne vibration implication in typical behavioral patterns present in *A. officinalis*, such as turn alternation, aggregation, and production of stridulations. The use of substrate vibrations in all these behavioral dynamics—likely involved in defense mechanisms of the species—might be useful to the single individual to anticipate danger, avoiding encountering the predator or other disturbance sources.

### The first evidence

The first observations and speculations of the possible involvement of surface-borne vibrations in communication and behavioral processes of invertebrates began several decades ago. The entomologist Ossiannilsson (1949) was one of the first to suggest that substrate-borne waves produced by a leafhopper reached another individual through the plant, and not through the air (Cocroft et al., 2014; Hill, 2012; Hill et al., 2019; Hill & Wessel, 2016). However, his suggestion on the use of this vibrational communication form remained almost ignored by the scientific community, until Strübing (1958) definitively demonstrated that this group of insects requires vibrational signals for mating (Cocroft et al., 2014; Hill et al., 2019; full translation in Strübing, 2014). Later, the possibility that females of *Drosophila persimilis* could perceive the courtship songs of males in the form of substrate-borne vibrations was suggested by Waldron (1964), speaking of “*a pulsed vibration sound*, page 191”. In the 1970s, clear evidence that substrate-borne vibrations were not only an artifact due to sound production but also the primary stimulus used in mating interactions—courtship and rivalry—by the cydnid bug *Tritomegas* was provided by Gogala and his group (1974) (Cocroft et al., 2014; Hill, 2012; Hill & Wessel, 2016). Shortly after that, other studies showed that vibrational communication in arthropods is not solely involved in courtship and mating. Research conducted on *Paruroctonus*—a sand scorpion—found that this arachnid uses substrate-borne vibrations, accidentally produced by prey, not only to detect prey but also to evaluate direction and distance from it, making the act of predation quicker and more effective (Brownell & Farley, 1979, 1984; Hill, 2012; Hill & Wessel, 2016).

Many other subsequent studies have indicated and demonstrated that invertebrates widely use vibrational communication for a vast range of behavioral processes of fundamental importance in the animal’s life—for survival and maintaining the species, as we illustrate in the next sections.

### Courtship, conspecific recognition, and mating

In animals, courtship consists of behavioral patterns—often ritualized and multimodal—concluding with mating aimed at reproduction and survival of the species (Alexander, Marshall, & Cooley, 1997; Ewer, 1968; Mitoyen, Quigley, & Fusani, 2019; Ota, Gahr, & Soma, 2015). Notably, in invertebrates, courtship plays an essential role in recognition of the species and sex of the partner and consists of actions leading to appropriate responses by the latter (Alexander et al., 1997; Ewer, 1968; Mitoyen et al., 2019; Ota et al., 2015). As we illustrate below, many studies have provided relevant evidence of the involvement of vibrational communication in conveying essential information to potential mates during the processes of courtship, conspecific recognition, and mating in arthropods, mainly in insects and arachnids. The information content is coded inside the temporal and spectral features of substrate-borne signals produced by the sender and/or receiver (Žunič, Virant-Doberlet, & Čokl, 2011). Several modes and characteristics concerning the use of substrate-borne vibrations were described based on species, but all appear involved in these intraspecific social behaviors.

The leafhopper *Scaphoideus titanus* Ball—grapevine specialist, and vector of the Flavescence dorée—uses substrate-borne vibrational signals for mate recognition and location (Mazzoni et al., 2009). Experimental observations demonstrated that, after being placed on a plant in the presence of females, males of *S. titanus* start spontaneously producing vibrational signals after a few minutes (Mazzoni et al., 2009). Following a response from females, males start putting in place a searching behavior. Otherwise, not receiving a reply, they stay stationary or jump off the plant (Mazzoni et al., 2009).

The planthopper *Hyalesthes obsoletus* Signoret has a mating behavior similar, in certain aspects, to that of *S. titanus*. Still, in this species, both sexes can start interacting through a vibrational call followed by a duet of recognition (Mazzoni, Lucchi, Ioriatti, Doberlet-Virant, & Anfora, 2010).

In the psyllid *Cacopsylla picta* Förster, 1848 as well, the pair formation process consisting of identification and courtship is based on vibrational communication (Oppedisano et al. 2020). Females start communicating by producing a series of vibrational pulses to identify males. If males reply, during the courtship, a duet is established through a set of prepulses and a “buzz” (Oppedisano et al., 2020).

In three different species of *Drosophila*—*D. suzukii*, *D. biarmipes*, and *D. melanogaster*—substrate-borne vibrations produced using locomotion, fluctuations of the abdomen, and thoracic wing muscles are different in both the repertoire and temporal and spectral parameters. Nevertheless, these vibrations are associated with courtship behaviors in all these species (Mazzoni, Anfora, & Virant-Doberlet, 2013).

In *Aacanthocnema dobsoni* Froggatt, 1903—a species able to communicate through substrate-borne vibrations—mating, calling behavior, and the females’ choice were investigated using playback experiments (Lubanga, Peters, & Steinbauer, 2016). Males of different sizes and ages varied in the production of substrate-borne vibrations, going from a lower dominant frequency for more significant-sized individuals to a higher intensity and pulse rate in the oldest individuals. Responses from females, however, were not influenced by body size or age of males, which often mated with unresponsive females instead of with virgin females responding to their calls. These psyllids thus seem to use substrate-borne vibrations for mate attraction, but not for mate selection (Lubanga et al., 2016).

Rather than in courtship, substrate-borne vibrations seem to play a role in the intraspecific communication at the level of intermale agonism in the New Zealand orthopter *Deinacrida rugosa*, when individuals are in a mixed-sex group (Howard, Schmidt, Hall, & Mason, 2018). In the southern green stink bug, *Nezara viridula*, the mechanisms guiding males in orientation and detection of the source of vibrational signals emitted by stationary females as directional cues were recently studied (Prešern, Polajnar, de Groot, Zorović, & Virant-Doberlet, 2018). The authors observed that males positioned their legs, provided of mechanoreceptors, on different sides of the plant branching, and that orientation at the branching point was not random (Prešern et al., 2018). Only a time delay of the vibrational signal between different legs stretched across the branching was a reliable directional cue because the amplitude of the signal at the branching point was frequently higher on the stalk away from the female (Prešern et al., 2018).

### Predation, defense strategies, and eavesdropping

In animal behavior, predation represents the act of capture and killing prey as a source of food (“Predation,” 1998), allowing transferring of energy from living animal to living animal (Minelli, 2008) and controlling the energy flux through the ecosystem (Simard & Harvey, 2010). Predation affects most aspects of the life of animals (e.g., foraging, mating, habitat selection). Hence, animals have developed many antipredator behaviors, such as vigilance and alarm calls, chemical defense, escaping, thanatosis, mimicry, and so on (Dugatkin, 2008; Endler, 1981; Gill & Bierema, 2013; Hill et al., 2019).

In such a complex, dynamic network of intraspecific and interspecific communications, eavesdropping plays an essential role in animal communication and survival mechanisms relative to predation and defense strategies (Alcock, 2009; Greenfield, 2010; Hill et al., 2019; Hill & Wessel, 2016; Krams, 2010; McGregor & Peake, 2000; Peake, 2005; Virant-Doberlet et al., 2019). Moreover, within this network of signaling, every single individual can act both as the sender or receiver and as the eavesdropper (Greenfield, 2010; Hill et al., 2019; Hill & Wessel, 2016; Sitvarin, Gordon, Uetz, & Rypstra, 2016). Among the other sensory systems (sight, hearing, touch, smell) used to communicate and intercept environmental signals, animals also possess highly sensitive receptors able to detect substrate vibrations (Virant-Doberlet et al., 2019). Table 1 illustrates the mechanoreceptors known in invertebrates (Keil, 1997, 2001).

Biotremology represents an emerging discipline, so some terms and definitions have not been wholly defined or accepted yet (Hill et al., 2019). For instance, according to the definition by Bradbury and Vehrencamp (1998), the term “cue” describes a nonevolving use of information by unintended receivers that do not change their behavior to increase the sender’s fitness (Hill et al., 2019). The predator–prey interactions are included in biotremology because of the intrinsic use of vibrational behavior, although predators and prey do not employ strategies that define a classical communication system (Hill et al., 2019). Predators can perceive prey through incidental vibrations in the substrate, and they have evolved their morphology and behavior to increase efficiency and success of capture (Hill et al., 2019). Similarly, prey have evolved their morphology and behavior to elude predators by detecting the incidental substrate vibrations produced by predators (Hill et al., 2019). This interpretation does not integrate well in the definition of “cue” as passively acquired information, and, in biotremology, scientists have currently been referring to the stimulus in these exchanges as cues (Hill et al., 2019).

Inside an “active space,” predators like the wolf spider (the eavesdropper) can intercept the substrate-borne signal component produced by a planthopper (the sender) that is establishing a vibrational communication with another planthopper (the receiver) (Hill, 2009; Hill & Wessel, 2016; Sitvarin et al., 2016). Conversely, the same predator can produce incidental substrate-borne vibrations with its body while moving on a surface, inadvertently alerting an unintended receiver of the potential threat, and allowing it to escape (Hill, 2009; Hill & Wessel, 2016; Sitvarin et al., 2016). Vibrations produced by predators are difficult to conceal and may be helpful in alerting prey of the imminent attack (Hill, 2009; Hill et al., 2019; Sitvarin et al., 2016). Furthermore, appropriateness of the response from the prey depends on its familiarity with the predator (coexistence or not over evolutionary time) (Hill et al., 2019; Sitvarin et al., 2016).

Many studies have demonstrated the involvement of vibrational behaviors inside this predation-eavesdropping-defense interchangeable cycle. Termites, generally preyed on by ants, mostly communicate through substrate-borne vibrations and use these to eavesdrop on ant vibrations (Oberst, Bann, Lai, & Evans, 2017). The termite species *Coptotermes acinaciformis* can detect its main predator—the ant species *Iridomyrmex purpureus—*only through the vibrational cues from walking, which are 100 times higher in ants than in termites (Oberst et al., 2017).

The stink bug *Podisus maculiventris* feeds on many kinds of prey, particularly lepidopteran defoliators (McPherson, 1982; Pfannenstiel et al., 1995). These insects can locate prey—for example, the green cloverworm, *Plathypena scabra—*using as a cue the substrate-borne vibrations produced by the latter when chewing on leaves (Pfannenstiel et al., 1995).

Some sand-dwelling invertebrates, such as desert scorpions and antlion larvae, use substrate-borne vibrations in predator–prey interactions (Brownell & Farley, 1979, 1984; Devetak, 2014; Kuszewska, Miler, Filipiak, & Woyciechowski, 2016; Mansell, 1996, 1999; Podlesnik, Klokočovnik, Lorent, & Devetak, 2019; Scharf, Lubin, & Ovadia, 2011). Experiments on the antlion species *Euroleon nostras* proved that vibrational stimuli produced by prey (*Lasius fuliginosus* ants) on the surface of the sand lead the antlions, which are located in deeper sand layers, to move towards the surface (Podlesnik et al., 2019).

A surprising form of mutualism mediated by substrate-borne vibrations was detected between some species of ants (*Crematogaster mimosa* and *Crematogaster sjostedti*) and the acacia tree (*Acacia zanzibarica*) where these insects live (Hager & Krausa, 2019; Hill, 2019). Ants feed on nonflower nectar sources of the acacia tree and live in its swollen nodules at the base of thorns (Hager & Krausa, 2019; Hill, 2019). In this form of mutualism, where both species have a reciprocal benefit, ants are recruited to defend the tree against herbivores through substrate-borne vibrations produced by the same predator and carried through the tree’s body (Hager & Krausa, 2019; Hill, 2019). Ants can distinguish the different types of substrate-borne vibrations through the host acacia tree, preparing to attack only in response to vibrations produced by herbivores (e.g., a goat), but not to waves generated by wind (Hager & Krausa, 2019; Hill, 2019). Ants can use substrate-borne vibrations generated by herbivores as long-distance alarm cues, and, importantly, they can use information from these vibrations to determine the direction to follow to attack the source of danger (Hager & Krausa, 2019; Hill, 2019).

#### Defense mechanisms and eavesdropping in *A. officinalis*

Terrestrial isopods have developed different behavioral strategies to defend against predators, including escape, acoustic warning, chemical secretions, specific postures (such as conglobation), and feigning death (Cazzolla Gatti, Messina, Tiralongo, Ursino, & Lombardo, 2019; Schmalfuss, 1984; Tuf, Drábková, & Šipoš, 2015; Witz, 1990). These behavioral patterns assist in increasing the fitness of single individuals inside the species, thus decreasing the probability of predation (Cazzolla Gatti et al., 2019). Commonly, different families of terrestrial isopods—for instance, Armadillidae and Armadillidiidae—use conglobation as a preferred defense strategy (Tuf et al., 2015).

We have found evidence suggesting that *A. officinalis* might use typical behavioral processes—that is, turn alternation (Cividini & Montesanto, 2018a, 2018b), aggregation (Cividini & Montesanto, 2018c), and stridulation (Cividini, Sfenthourakis, & Montesanto, 2020)—as potential defense strategies against predators. These behavioral dynamics, mediated by substrate vibrations, might also allow individuals to anticipate and avoid disturbance and injury before encountering the sources of them. In the succeeding paragraphs, we illustrate our findings.

##### Turn alternation

An increase in the phenomenon of turn alternation is a natural mechanism, present in various species of terrestrial isopods, to react against a particularly unfavorable condition—mainly, for example, food deprivation (Hughes, 1978), disturbance on the substrate (Houghtaling & Kight, 2006), signals from indirect predators (Hegarty & Kight, 2014), or exposure to predators (Carbines, Dennis, & Jackson, 1992; Hughes, 1967, 1978). An increased number of alternating turns in animals was also observed by Hughes (1967) following their exposure to excessive light or to dry environments, and by Ono and Takagi (2006) following the artificial stimulation of animals.

**Fig. 3:**
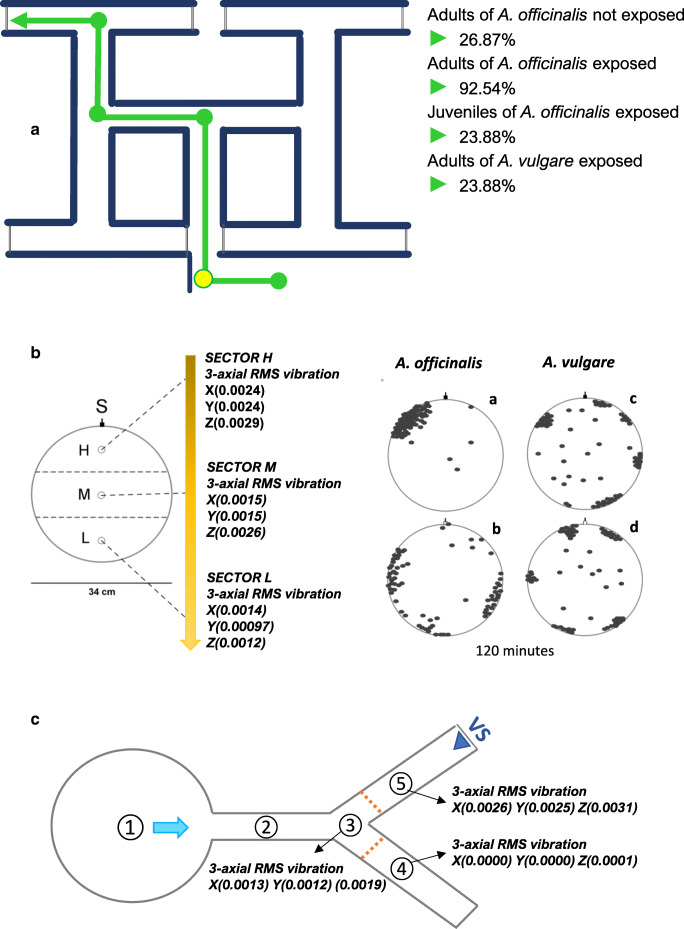
**a** Diagram of the test apparatus (T-maze) used to test turn alternation patterns relative to the presence of non-specific substrate-borne vibrations in adults and juveniles of *A. officinalis* and adults of *A. vulgare*. Every animal was forced to turn right (yellow dot) before entering the labyrinth. The green path corresponds to three correct turn alternations. The percentage of animals that followed the green path is reported per group. b Diagram of the test apparatus (arena) used to test aggregation patterns relative to the presence of nonspecific substrate-borne vibrations in adults of *A. officinalis* and *A. vulgare*. The arrow indicates the progressive decrease in the vibrational intensity from Sector H to Sector L. On the right, the dynamics of aggregation of *A. officinalis* and *A. vulgare* after 120 minutes in the absence (a and c) and presence (b and d) of nonspecific substrate-borne vibrations, respectively. c Diagram of the test apparatus (Y shape) used to test directional patterns relative to the presence of nonspecific substrate-borne vibrations, or species-specific stridulations in adults of *A. officinalis.* 1 = start point; 2 = hallway; 3 = forking; 4 = branch without vibrations; 5 = branch with vibrations; VS = vibrational source. RMS vibration = Root-mean-square amplitude of the vibration data about zero. X, Y, Z = cartesian axes. The characteristics of nonspecific substrate-borne vibrations, used for all experiments, are illustrated in Fig. 4. The features of species-specific stridulations are shown in Fig. 5. (Color figure online)

Intending to understand which other factors and physiological mechanisms may be involved in the increase of alternating turns in terrestrial isopods, we investigated changes in the pattern of turn alternation in adult individuals of *A. officinalis* following exposure to substrate-borne vibrations (see Fig. 3a) (Cividini & Montesanto, 2018a). Additionally, *A. officinalis* was also compared with a nonstridulating species—*Armadillidium vulgare*—and with juvenile conspecifics, both of which were exposed to substrate-borne vibrations (Cividini & Montesanto, 2018a, 2018b).

**Fig. 4:**
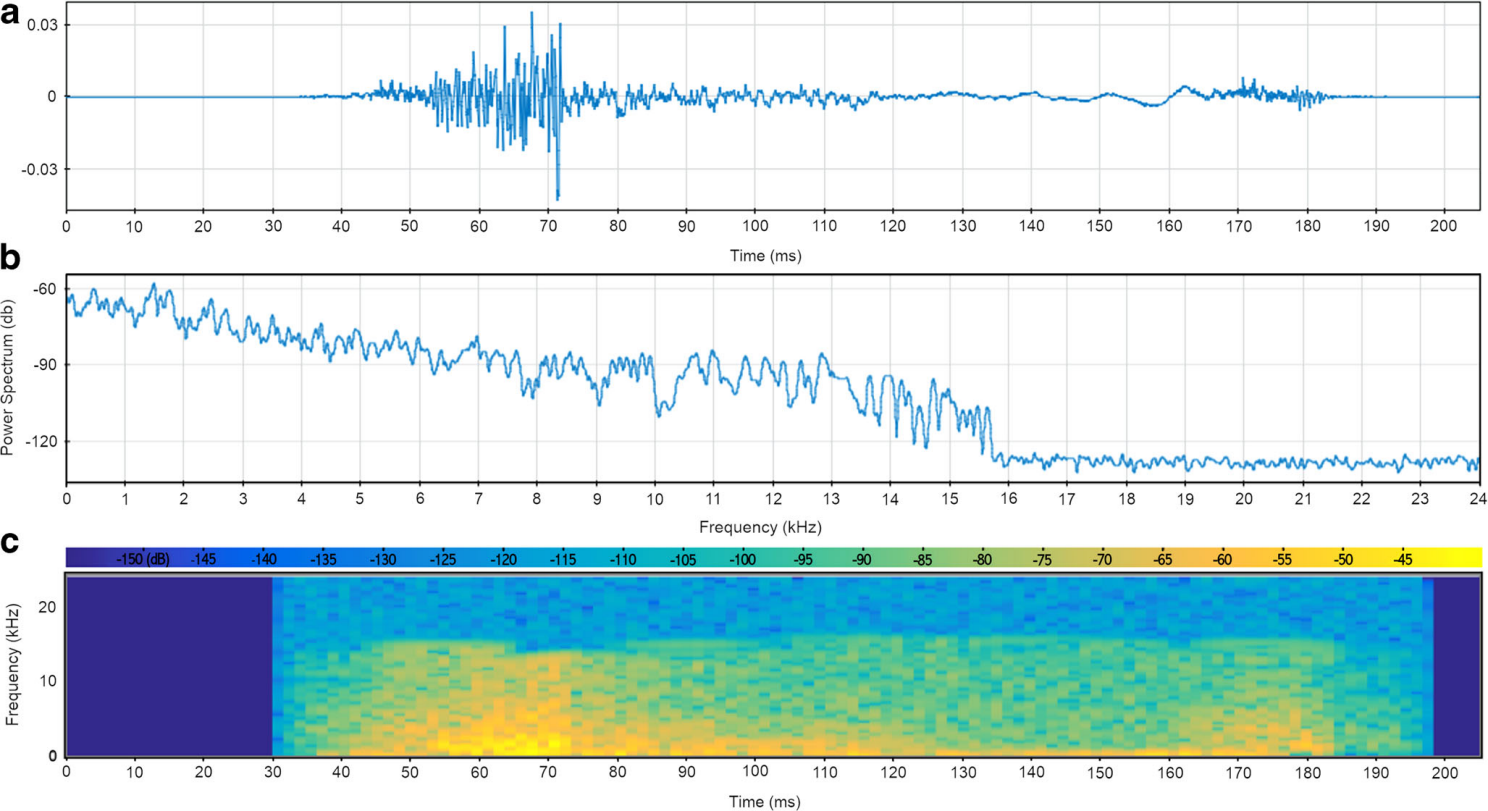
Nonspecific substrate-borne vibrations generated with software. a *Oscillogram*—view of the signals in the time domain. b *Spectrum*—view of the frequency spectrum of the signals. c *Spectrogram*—view of the signals in the time-frequency domain. The spectrogram of a nonstationary signal is an estimate of the time evolution of its frequency content. The color bar indicates the power of the short-time Fourier transform in decibels—yellow colors are frequencies with a higher power, and blue colors are frequencies with very low power. The graphs were created using the Signal Analyzer App in MATLAB R2018b (9.5) (The MathWorks Inc., Natick, MA, USA). From Cividini et al. (2020; http://creativecommons.org/licenses/by/4.0/). (Color figure online)

Random nonspecific substrate-borne waves were artificially produced through software to simulate those present in a natural environment—at the level of potential active space—in both amplitudes and frequencies (see Fig. 4). As previously described, the vibrational signal pattern is quite irregular, with a nonmonotonic decreasing of amplitude (Čokl 1988; Čokl et al., 2007; Mazzoni et al., 2014). These signals are nonstationary because their frequency content changes with time, as illustrated by the spectrogram that represents an estimate of the time evolution of the signal’s frequency content (see Fig. 4c).

Our results pointed out a statistically significant association between the behavioral dynamics of adults of *A. officinalis* and the exposure to substrate-borne vibrations, species (Cividini & Montesanto, 2018a), and age of individuals (Cividini & Montesanto, 2018b). Adults of *A. officinalis* are sensitive and reactive to the presence of substrate-borne waves, and, at the parity of exposure level, significantly increase the number of alternating turns carried out compared with adults of *A. vulgare* and juvenile conspecifics (Cividini & Montesanto, 2018a, 2018b). Moreover, *A. officinalis*’s capability of perceiving and reacting to substrate-borne vibrations in terms of an increased turn alternation appears to improve with age (Cividini & Montesanto, 2018b).

The presence of substrate-borne waves might be interpreted as a source of disturbance or imminent danger, leading animals to increase turn alternation to escape from unfavorable environmental conditions (Cividini & Montesanto, 2018a, 2018b). Thanks to its ability to produce stridulations, *A. officinalis* might perceive, utilize, and manage substrate-borne vibrations better than other nonstridulating species and for multiple aims (Cividini & Montesanto, 2018a, 2018b; Cividini et al., 2020)—for example, defense mechanisms (also through eavesdropping) and intraspecific and interspecific communication.

##### Aggregation

In terrestrial isopods, the phenomenon of aggregation among conspecifics is well known, although why it happens, as well as the mechanisms favoring it, have not yet been completely clarified. Aggregation is thought to be a way to prevent dehydration following a loss of water through the gills on pleopods (appendages attached to abdomen), on the ventral part of the pereion (thorax), and on the dorsal surface, because the cuticle of these animals contains many pores and low lipid content (Allee, 1926; Brockett & Hassall, 2005; Broly et al., 2014; Edney, 1951, 1954, 1968; Hadley & Quinlan, 1984; Quinlan & Hadley, 1983). Moreover, aggregation could serve as a defense mechanism (Broly et al., 2013; Ims, 1990; Schmalfuss, 1984), as well as favoring coprophagy (Broly et al., 2013; Hassall & Rushton, 1982; Hassall, Tuck, & James, 2005), reproduction, and reduction of oxygen consumption (Allee, 1926; Broly et al., 2013; Takeda 1984). An involvement of aggregation pheromones was proposed (Broly et al., 2012), but other, still unknown factors might also be involved in favoring or disadvantaging this phenomenon.

One of the projects developed by our research group was thus to investigate the possible influence of the presence of substrate-borne waves on aggregation phenomena in terrestrial isopods. The aggregative behavior of *A. officinalis*—sensitive to substrate-borne vibrations and able to produce stridulations—was compared with that of *A. vulgare*, a species without stridulatory apparatus (Cividini & Montesanto, 2018c). As shown in Fig. 3b, for each of the two species, a group of 73 individuals exposed to nonspecific substrate-borne vibrations (see Fig. 4) was tested compared with a nonexposed control group of the same size. Vibrational intensity progressively decreased from Sector H to Sector L (see Fig. 3b). Data on behavioral patterns were recorded after 120 minutes (Cividini & Montesanto, 2018c).

Consistent with the results obtained for turn alternation (Cividini & Montesanto, 2018a), and, unlike *A. vulgare*, individuals of *A. officinalis* significantly react to the presence of substrate-borne vibrations, moving away from the zones with higher vibrational intensity (Cividini & Montesanto, 2018c). Moreover, in the presence of substrate-borne waves, the capability of *A. officinalis* to form a unique, large aggregate appears reduced, as if animals have a lower ability to localize their conspecifics inside the arena. That speculation might explain the presence of a higher number of aggregates and isolated subjects than in the absence of substrate vibrations (Cividini & Montesanto, 2018c).

As with some species of insects (Castellanos & Barbosa, 2006; Cocroft, 2001; Evans et al., 2009; Hager & Krausa, 2019; Hill, 2019; Oberst et al., 2017), *A. officinalis* might be able to distinguish—quantitatively and qualitatively—substrate-borne vibrations produced by its conspecifics inside an aggregate compared with nonspecific waves from the environment (Cividini & Montesanto, 2018c). Nonspecific substrate-borne waves might thus play a double role—namely, acting as an alarm and interfering with a hypothetical capability to use species-specific substrate-borne waves, as a possible “call” to aggregation (Cividini & Montesanto, 2018c). However, unlike other, more studied invertebrates, no information on the existence of mechanoreceptors or acoustic receptors exists in terrestrial isopods, which prevents definite conclusions. Further studies are needed in this direction.

##### Stridulation

The ability of insects to produce acoustic and vibratory signals as possible forms of intraspecific and interspecific communication is well known. Among the other aims, this ability is used to interact with conspecifics, obtain information from the surrounding environment, and defend against predators. Insects produce sounds in five different ways, using particular bodily structures—namely, by stridulation, by percussion, by vibration, by using particular membranes called tymbals, and by forcibly ejecting air or fluid (Alexander, 1957; Ewing, 1989). Moreover, insects can produce and modulate sounds in a targeted way according to specific needs and situations (Alexander, 1957)—for instance, by using species-specific songs for recognizing and locating mates, and by using nonspecific songs to obtain information from the environment regarding dangers, rivals, or predators (Čokl & Virant-Doberlet, 2003).

**Fig. 5:**
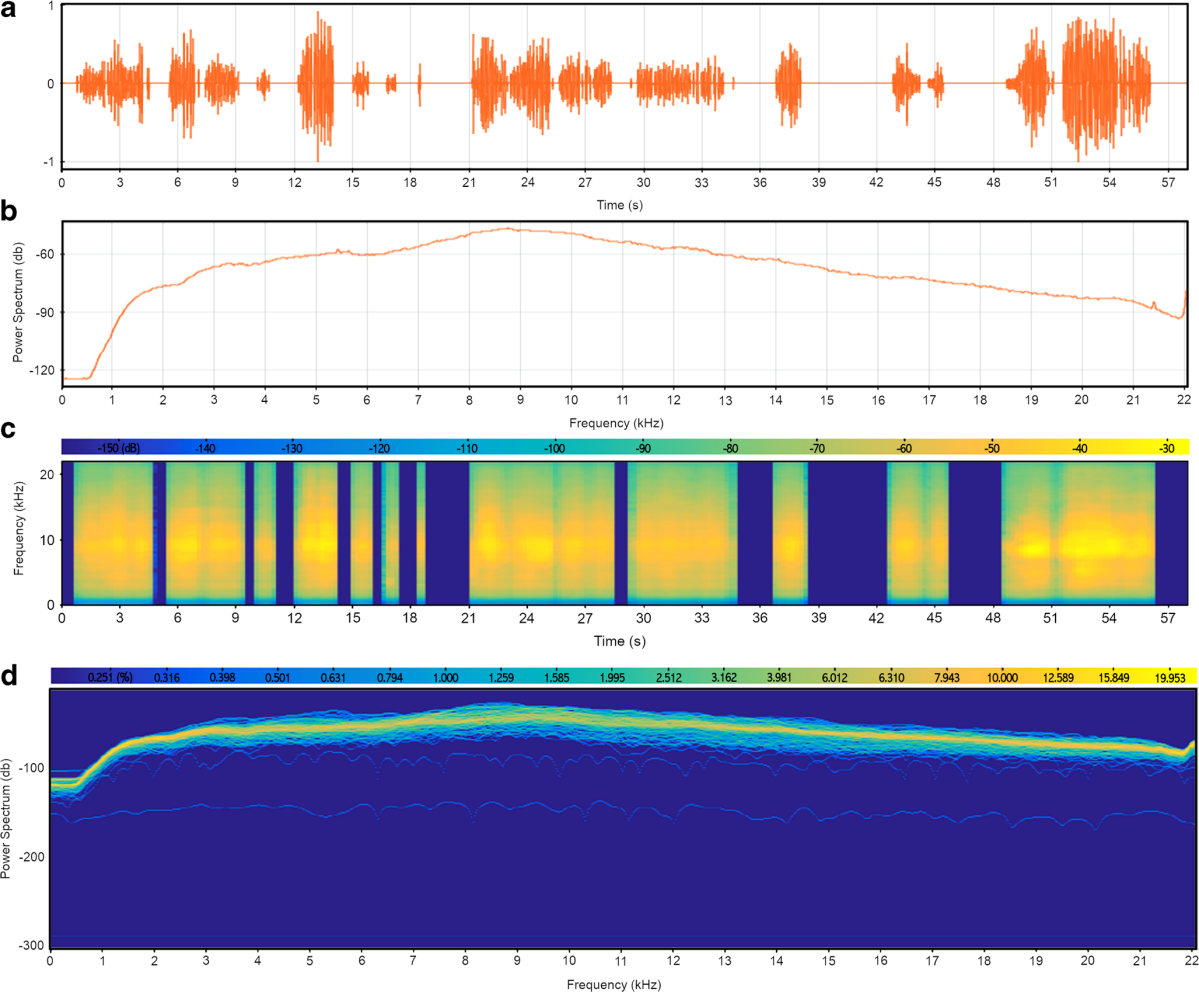
**S**tridulations of *Armadillo officinalis* during conglobation, a potential form of secondary defense against predation. **a** *Oscillogram*—view of signals in the time domain. **b** *Spectrum*—view of the frequency spectrum of the signals (highest intensity reached around 9 kHz). **c** *Spectrogram*—view of the signals in the time-frequency domain. The spectrogram of a nonstationary signal is an estimate of the time evolution of its frequency content. The color bar indicates the power of the short-time Fourier transform in decibels—yellow colors are frequencies with a higher power, and blue colors are frequencies with very low power. The strong yellow horizontal line shows the existence of a 9-kHz tone in all the stridulation sets. **d** *Persistence spectrum*—a time-frequency view that shows the percentage of the time that a given frequency is present in the signal. The graphs were created using the Signal Analyzer App in MATLAB R2018b (9.5) (The MathWorks Inc., Natick, MA, USA). From Cividini et al. (2020; http://creativecommons.org/licenses/by/4.0/). (Color figure online)

Some terrestrial isopods of the roller type, like *A. officinalis*, have the same capability of producing stridulations and vibrations. However, they have not received as much attention as other arthropods. Based on information from our previous studies, we investigated whether species-specific stridulations produced by *A. officinalis* during conglobation triggered by predators (see Fig. 5) could be perceived by a nearby conspecific (the eavesdropper) as an alert cue, thus potentially representing a possibility to anticipate danger. In the same experiment, we further deepened the capability of perceiving nonspecific substrate-borne vibrations (see Fig. 4) by *A. officinalis*.

Our results demonstrated a significant shift of *A. officinalis* away from the vibrational source with both types of vibrations used, mainly choosing the branch of the test apparatus without vibrations (see Fig. 3c). This suggests that animals interpret both species-specific stridulations and nonspecific substrate-borne waves as a source of potential disturbance or danger (Cividini et al., 2020).

*Armadillo officinalis* can produce stridulations only when it assumes the typical ball shape during conglobation, a mechanism mainly considered as a potential antipredator strategy (Caruso & Costa, 1976; Cazzolla Gatti et al., 2019; Schmalfuss, 1984; Taiti et al., 1998; Tuf et al., 2015; Witz, 1990). As with many species of insects (Kowalski, Lakes-Harlan, Lehmann, & Strauß, 2014; Masters, 1979, 1980), production of sounds during conglobation could be a secondary form of defense based on an acoustic warning, and used by *A. officinalis* to deter a predator following contact (Cividini et al., 2020). For this reason, conspecifics might interpret species-specific stridulations as an alarm signal, moving away from the source of disturbance (Cividini et al., 2020). The high sensitivity to nonspecific substrate-borne vibrations has been further demonstrated and might provide *A. officinalis* with a better chance of survival thanks to the ability to anticipate dangers and adverse conditions (Cividini et al., 2020).

### Foraging and parental care

Foraging is the means through which animals acquire energy and nutrients to survive, grow, and reproduce (Kramer, 2001). An animal can directly consume food (feeding), store it (hoarding), or give it to other individuals (provisioning) (Kramer, 2001).

In herbivorous insects living in groups, communication via substrate-borne vibrations might help them to find food, locate conspecifics, remain in the group, and avoid predation (Cocroft, 2001). Antlion larvae (Neuroptera: Myrmeleontidae) can use vibrational cues for modifying their foraging strategies and distinguishing prey of different sizes. In this way, these sedentary animals can save resources by ignoring smaller prey in favor of larger and energetically more advantageous prey (Kuszewska et al., 2016).

“*Parental care can be defined as any non-genetic contribution by a parent that increases the fitness of offspring, and can occur before or after laying or birth*” (Stahlschmidt & DeNardo, 2011). In nonmammals, parental care is likely regulated by hormones and consists of a wide diversity of systems and behaviors that have evolved multiple, independent times (Adkins-Regan & Smiley, 2019). In invertebrates, many forms of parental care exist. Among these, the most basic types are the use of trophic eggs (no direct maternal–offspring contact) and lingering near eggs and offspring to offer modest protection from predators or parasitoids (Trumbo, 2012).

Studies by Cocroft (1996, 1998, 1999) uncovered the existence of a complex mechanism of communication mediated by substrate-borne vibrations between nymphs and parents in a subsocial treehopper (*Umbonia crassicornis*), in response to natural predators, such as wasps. In the absence of predators, nymphs produce few synchronized signals. Still, these signals significantly increase in the presence of a wasp, to inform the mother about the side of the aggregation wherein the threat is more imminent (Cocroft 1996, 1998, 1999; Ramaswamy & Cocroft, 2009; Trumbo, 2012). Females, nevertheless, often respond to the presence of predators by buzzing potential threats through their elongated pronotum before nymph signaling, which indicates that nymphs’ signals have a double aim—namely, influencing the mother’s behavior and alerting her (Cocroft 1996, 1998, 1999; Ramaswamy & Cocroft, 2009; Trumbo, 2012). Only after the predator departs does the mother start emitting high rate signals (Cocroft, 1999).

## Discussion

Vibrational communication is undoubtedly one of the most ancient and widespread forms of animal communication. It involves many taxa and implies the use of ubiquitous receptors (Cocroft et al., 2014; Hill, 2001, 2008, 2012; Hill et al., 2019; Virant-Doberlet, 2019). From an ecological context, vibrational communication thus has a relevant place with a long evolutionary history, likely evolving along with chemical communication in the early Metazoa (Endler, 2014; Hill et al., 2014; Virant-Doberlet, 2019).

An increasing number of studies have demonstrated how this way of communicating is part of a complex, dynamic network of intraspecific and interspecific signaling, in which conspecifics, heterospecifics, rivals, and exploiters are actively involved in information exchanges (Cocroft et al., 2014; Cocroft & Rodríguez, 2005; Hill et al., 2019; McVean & Field, 1996; Stewart & Zeigler, 1984; Virant-Doberlet et al., 2014; Virant-Doberlet et al., 2019). Vibrational communication is not a private, short-range, highly specialized communication channel with limited use, as was previously thought, compared with acoustic communication, and it is not free from eavesdropping (Hill et al., 2019; Virant-Doberlet et al., 2019). Indeed, as illustrated previously, this communication modality is widely used by animals to manage vital, essential behavioral processes relative, for instance, to reproduction, predator–prey interaction, foraging, and parental care.

Biotremology is a new, emerging discipline dealing with the study of vibrational communication, and, because of its peculiar features, it cannot be accommodated inside bioacoustics (Hill et al., 2019). Compared with air-borne communication, substrate-borne vibrational communication occurs in a more complex and unpredictable space (Hill et al., 2019; Virant-Doberlet et al., 2019). Sounds travel through a homogeneous enough medium, such as air or water. Conversely, substrate-borne vibrations travel through heterogeneous substrates, with different physical properties that can limit the effective range of the vibrational component (Hill et al., 2019; Virant-Doberlet et al., 2019). Still, signals produced by animals have adapted to their lived environment and to the sensitivity of their receptors concerning frequencies used (Hill et al., 2019; Virant-Doberlet et al., 2019). Furthermore, sounds and vibrational signals are perceived differently—through ears or mechanoreceptors, respectively—and are elaborated in different parts of the nervous system (Hill et al., 2019; Strauß & Stumpner, 2015; Stritih & Stumpner, 2009; Virant-Doberlet, Čokl, & Zorovic, 2006).

Behaviors, and knowledge derived from these behaviors, are considered differently in biotremology compared with traditional ways based on sound communication (Hill et al., 2019). Predator–prey interaction and rapid hatching are included and studied in biotremology because of the intrinsic use of vibrational behavior, despite being outside the classical definition of the communication paradigm (Hill et al., 2019). However, this new knowledge from outside the communication paradigm can be used inside the paradigm itself (Hill et al., 2019). In predator–prey interactions, both predators and prey may perceive the substrate-borne vibrations reciprocally produced in an incidental mode. So, they have evolved and coevolved morphology and behavior to increase the probability of succeeding in predation or in eluding the predator (Hill et al., 2019). This modality is not part of classical communication, nor does it fit the passive definition of “cue.” Predators and prey both respond to information in a way that benefits the receiving individual because both serve as receivers in the interaction (Hill et al., 2019). Furthermore, if encounters between a predator and prey are frequent, and not rare events, then natural selection might act positively or negatively on both, and behavior will evolve (Hill et al., 2019).

Similarly, the studies on rapid hatching have revealed the capability to discriminate between substrate-borne incidental cues and waveforms from rain, wind, or other environmental events (Hill et al., 2019; Warkentin, 2005; Warkentin, Caldwell, & McDaniel, 2006). These mechanisms might be somewhat common and used within the classical communication paradigm (Hill et al., 2019).

### Future research on terrestrial isopods: Perspectives and new studies

Most of the studies regarding vibrational communication in arthropods have focused on insects and arachnids as the best animal models, and so, for these taxa, much evidence of its use as a source of information from the surrounding environment exists.

Nevertheless, other taxa—never considered before—have suited features to be used as behavioral models in the study of vibrational communication mechanisms. Some terrestrial isopods of the roller type are among these.

The study of communication mechanisms mediated by surface-borne vibrations in *A. officinalis* and other isopod species with similar characteristics might offer broad-spectrum insights on this kind of communication in arthropods. Indeed, information from studies of new taxa may contribute to integrating and better elucidating already known information from more studied species, confirming how vibrational communication is essential and central in invertebrate behaviors and interactions.

Our future efforts will thus explore further aspects of vibratory communication as a form of intraspecific and interspecific signaling and exchange of information in terrestrial isopods. Also, we will consider other stridulating species besides *A. officinalis*.

## Electronic supplementary material

ESM 1 (DOCX 23 kb)
