# Supplementary material for: Biotremology in arthropods
Source: Learn Behav. 2020 Jul 6;48(3):281–300. doi: 10.3758/s13420-020-00428-3 (PMC7473968; doi:10.3758/s13420-020-00428-3)
Supplement: Supplementary file 1 — (DOCX 23 kb) [file 13420_2020_428_MOESM1_ESM.docx]

**Biotremology in arthropods**

Sofia Cividini^a^ and Giuseppe Montesanto^b^

*^a^Department of Biostatistics, University of Liverpool, Liverpool, UK*

*^b^Dipartimento di Biologia, Università degli Studi di Pisa, Pisa, Italy*

**Table 1: References**

Autrum, H. (1942). Schallempfang bei Tier und Mensch. *Naturwissenschaften*, 30, 69–85. <https://doi.org/10.1007/BF01475622>

Autrum, H., & Schneider, W. (1948). Vergleichende Untersuchungenüber den Erschütterungssinn der Insekten. *Zeitschrift für vergleichende Physiologie*, 31, 77–88. <https://doi.org/10.1007/BF00333879>

Bareth, C., & Juberthie-Jupeau, L. (1986). Ultrastructure des trichobothries de l’antenne de *Campodea kervillei* Denis et *Plusiocampa cognata* Conde´ (Apterygota: Diplura: Campodeidae). *International Journal of Insect Morphology and Embryology*, 15, 429–438. <https://doi.org/10.1016/0020-7322(86)90035-8>

Berg, J. (1994). Feinstruktur und Ha¨utung der Sensillen auf der Antennengeissel von *Lepisma saccharina* Linnaeus (Zygentoma) und *Machilis spec.* (Archaeognatha). Dissertation, Universität Karlsruhe.

Camhi, J. M. (1980). The escape system of the cockroach. *Scientific American*, 243(6), 144–156. <https://www.jstor.org/stable/24966482>

Christian, U. (1971). Zur Feinstruktur der Trichobothrien der Winkelspinne *Tegenaria derhami* (Scopoli), (Agelenidae, Araneae). *Cytobiologie*, 4, 172–185.

Drašlar, K. (1973). Functional properties of trichobothria in the bug *Pyrrhocoris apterus* (L.). J. *Journal of comparative physiology volume*, 84, 175–184. <https://doi.org/10.1007/BF00697605>

Gaffal, K.P. (1976). The stimulus transmitting apparatus in the trichobothria of the bugs *Pyrrhocoris apterus* L. and *Dysdercus intermedius* (Dist.) and its influence on the dynamic of excitation in these sensilla. *Experientia*, 32, 166–168. <https://doi.org/10.1007/BF01937744>

Gnatzy, W. (1976). The ultrastructure of the thread-hairs on the cerci of the cockroach *Periplaneta* *americana* L.: The intermoult phase. *Journal of Ultrastructure Research*, 54, 124–134. <https://doi.org/10.1016/S0022-5320(76)80015-9>

Görner, P. (1965). A proposed transducing mechanism for a multiplyinnervated mechanoreceptor (Trichobothrium) in spiders. *Cold Spring Harbor Symposia on Quantitative Biology*, 30, 69–73.

Görner, P., & Andrews, P. (1969). Trichobothrien, ein Ferntastsinnesorgan bei Webspinnen (*Araneen*). *Z. vergl. Physiologie*, 64, 301–317. https://doi.org/10.1007/BF00340548

Gray, E.G. (1960). The fine structure of the insect ear. *Philosophical Transactions of the Royal Society of London. Series B, Biological Sciences*, 243, 75–94. <https://www.jstor.org/stable/2992598>

Haupt, J. (1970). Beitrag zur Kenntnis der Sinnesorgane von Symphylen (Myriapoda). I. Elektronenmikroskopische Untersuchung des Trichobothriums von Scutigerella immaculata Newport. *Zeitschrift für Zellforschung und Mikroskopische Anatomie*, 110, 588–599. <https://doi.org/10.1007/BF00330106>

Haupt, J. (1978). Ultrastruktur der Trichobothrien von Allopauropus (Decapauropus) (Pauropoda). Abh. Verh. Naturwiss. Verh. Hamburg, 21/22, 271–277.

Hoffmann, C. (1967). Bau und Funktion der Trichobothrien von Euscorpius carpathicus L. Z. Vergl. Physiol., 54, 290–352.

Lindauer, M., & Nedel, J.O. (1959). Ein Schweresinnesorgan der Honigbiene. *Zeitschrift für vergleichende Physiologie*, 42, 334–364. <https://doi.org/10.1007/BF00298125>

McIver, S.B., & Siemicki, R. (1984). Fine structure of antennal mechanosensilla of adult *Rhodnius prolixus* stål (Hemiptera: Reduviidae). *Journal of Morphology*, 180, 19–28. <https://doi.org/10.1002/jmor.1051800104>

Messlinger, K. (1987). Fine structure of scorpion trichobothria (Arachnida, Scorpiones). *Zoomorphologie*, 107, 49–57. <https://doi.org/10.1007/BF00312129>

Pix, W., Nalbach, G., & Zeil, J. (1993). Strepsipteran forewings are haltere-like organs of equilibrium. *Naturwissenschaften*, 80, 371–374. <https://doi.org/10.1007/BF01138795>

Risler, H. (1977). The construction of the auditory organ in male mosquitoes. *Fortschr. Zool.*, 24, 143–147.

Smola, U. (1970). Untersuchung zur Topographie, Mechanik und Stro¨mungsmechanik der Sinneshaare auf dem Kopf derWanderheuschrecke *Locusta migratoria*. *Zeitschrift für vergleichende Physiologie,* 67, 382–402. <https://doi.org/10.1007/BF00297907>

Tautz, J. (1977). Reception of medium vibration by thoracal hairs of caterpillars of *Barathra brassicae* L. (Lepidoptera, Noctuidae). I. Mechanical properties of the receptor hairs. *Journal of Comparative Physiology*, 118, 13–31. <https://doi.org/10.1007/BF00612334>

Tautz, J. (1978). Reception of medium vibration by thoracal hairs of caterpillars of *Barathra brassicae* L. (Lepidoptera, Noctuidae). II. Response characteristics of the sensory cell. *Journal of Comparative Physiology*, 125, 67–77. <https://doi.org/10.1007/BF00656832>

Thurm, U. (1965). An insect mechanoreceptor. I. Fine structure and adequate stimulus. *Cold Spring Harbor Symposia on Quantitative Biology*, 30, 75–82.

Voelker, W. (1982). Lebendbeobachtungen an kutikulären Reizübertragungsstrukturen campaniformer Sensillen und Hochauflösungs- Elektronenmikroskopie der reizaufnehmenden Sinneszellregion. Dissertation, WestfälischeWilhelms- Universität,Münster.
